# Simultaneous bilateral total ankle replacement using a 3-component prosthesis: Outcome in 26 patients followed for 2–10 years

**DOI:** 10.3109/17453674.2011.623570

**Published:** 2011-11-25

**Authors:** Alexej Barg, Heath B Henninger, Markus Knupp, Beat Hintermann

**Affiliations:** ^1^Clinic of Orthopaedic Surgery, Kantonsspital Liestal, Liestal, Switzerland; ^2^Department of Orthopaedics, Harold K Dunn Orthopaedic Research Laboratory, University of Utah, Salt Lake City, UT, USA

## Abstract

**Background and purpose:**

Total ankle replacement is an established surgical procedure in patients with end-stage ankle osteoarthritis. We analyzed complications and medium-term results in patients with simultaneous bilateral total ankle replacement.

**Patients and methods:**

10 women and 16 men, mean age 60 (SD 13) years, were followed for a median of 5 (2–10) years.

**Results:**

There were no intraoperative or perioperative complications, with the exception of 1 patient with prolonged wound healing. Major revision surgery was necessary in 6 of the 52 ankles, including 4 revisions of prosthetic components. The average pain score decreased from 6.9 (4−10) to 1.8 (0−4) points. The American Orthopaedic Foot and Ankle Society hindfoot score increased from 32 (SD 14) points preoperatively to 74 (SD 12) points postoperatively. The average range of motion increased from 28° (SD 12) preoperatively to 38° (SD 9) postoperatively. All 8 categories of SF-36 score improved.

**Interpretation:**

Simultaneous bilateral total ankle replacement is a suitable method for restoration of function and attainment of pain relief in patients with bilateral end-stage ankle osteoarthritis. The results of this procedure, including complication rates, revision rates, and functional outcome, are comparable to those reported in patients with unilateral total ankle replacement.

Total ankle replacement (TAR) is an established surgical procedure in patients with severe ankle osteoarthritis (Gougoulias et al. 2010), and is becoming an increasingly recommended treatment option instead of ankle arthrodesis (Saltzman et al. 2009). Several national arthroplasty registers have reported favorable medium-term results (Fevang et al. 2007, Henricson et al. 2007, Hosman et al. 2007, Skytta et al. 2010).

In patients with bilateral knee or hip osteoarthritis, simultaneous bilateral joint replacement has been reported to be a safe procedure (Kim et al. 2009a, b, Tsiridis et al. 2008). However, TAR differs from arthroplasties of the knee or hip joint, especially regarding indications, difficulty of surgical technique, and survivorship of prosthesis components (Rydholm 2007). There is a limited amount of literature on medium-term results in patients who have undergone simultaneous bilateral TAR (Barg et al. 2010b, Karantana et al. 2010a). In a previous study, we reported the 2-year outcome in 23 of the 26 patients reported in the current study (Barg et al. 2010b).

We performed this study to determine (1) the intraoperative and postoperative complication rate, including the need for surgical revision, (2) the degree of pain relief postoperatively, and (3) postoperative functional outcome including range of motion, quality of life, and level of activity.

## Patients and methods

The subset of patients in this study (patients with severe bilateral ankle arthropathy who underwent simultaneous bilateral TAR) was part of a larger prospective study group involving all patients who underwent TAR at our institution. Between June 2000 and October 2008, the senior author (BH) performed 52 TARs in 26 patients (mean age 60 (SD 13) years, 16 men) with bilateral, painful, immobilizing ankle arthritis. Preoperative diagnosis was rheumatoid osteoarthritis in 24 ankles, posttraumatic osteoarthritis in 18 ankles (15 ankles with ligamentous posttraumatic ankle osteoarthritis and 3 ankles with a history of lower leg fracture), gouty osteoarthritis in 6 ankles, hemophilic arthropathy in 2 ankles, and osteoarthritis due to hereditary hemochromatosis in 2 ankles (Table 1). All patients were followed for mean 5.2 (2–10) years. 2-year follow-up of 23 ankles has been reported previously (Barg et al. 2010b).

**Table 1.** Description of surgery in 26 patients with simultaneous bilateral TAR

Patient no.	Case no.	Side	Diagnosis	Tourniquet time (min)	Operative time (min)	Additional surgery
1	1	left	rheumatoid	71	114	
	2	right	rheumatoid	57		
2	3	left	posttraumatic	55	125	PATL **^b^**
	4	right	posttraumatic	80		PATL **^b^**
3	5	left	rheumatoid	88	169	
	6	right	rheumatoid	102		
4	7	left	rheumatoid	84	170	
	8	right	rheumatoid	107		
5	9	left	hemophilia	112	191	PATL **^b^**
	10	right	hemophilia	103		PATL **^b^**
6	11	left	gout	86	177	
	12	right	gout	113		
7	13	left	rheumatoid	95	181	PATL **^b^**
	14	right	rheumatoid	103		PATL **^b^**
8	15	left	rheumatoid	68	116	PATL **^b^**
	16	right	rheumatoid	59		PATL **^b^**
9	17	left	posttraumatic	89	155	lateral lig. reconstruction
	18	right	posttraumatic	91		lateral lig. reconstruction
10	19	left	rheumatoid	75	119	
	20	right	rheumatoid	61		
11	21	left	gout	101	193	MDCO **^c^**, double-hindfoot ADd, lateral lig. reconstruction
	22	right	gout	116		MDCO **^c^**, MTP-I arthrodesis, peroneus longus to peroneus brevis tendon transfer, lateral lig. reconstruction
12	23	left	hemochromatosis	69	173	
	24	right	hemochromatosis	125		supramalleolar osteotomy, PATL **^b^**
13	25	left	gout	121	213	subtalar arthrodesis, lateral lig. reconstruction
	26	right	gout	118		subtalar arthrodesis
14	27	left	posttraumatic	59	110	
	28	right	posttraumatic	65		
15	29	left	rheumatoid	78	136	
	30	right	rheumatoid	69		
16	31	left	rheumatoid	66	168	
	32	right	rheumatoid	123		re-arthrodesis talonavicular, tibialis anterior tendon reconstruction
17	33	left	posttraumatic	72	121	PATL**^b^**
	34	right	posttraumatic	64		
18	35	left	rheumatoid	110	209	medial lig. reconstruction
	36	right	rheumatoid	125		
19	37	left	posttraumatic	106	187	MDCO **^c^**, lateral lig. reconstruction
	38	right	posttraumatic	101		MDCO **^c^**
20	39	left	posttraumatic	89	164	dorsiflexion osteotomy of first metatarsal, peroneus longus to peroneus brevis tendon transfer, lateral lig. reconstruction, PATL **^b^**
	40	right	posttraumatic	95		dorsiflexion osteotomy of first metatarsal, peroneus longus to peroneus brevis tendon transfer, lateral lig. reconstruction
21	41	left	rheumatoid	94	172	double-hindfoot AD **^d^**, PATL **^b^**
	42	right	rheumatoid	99		double-hindfoot AD **^d^**, PATL **^b^**
22	43	left	posttraumatic	124 **^a^**	232	lateral lig. reconstruction
	44	right	posttraumatic	137 **^a^**		peroneus longus to peroneus brevis tendon transfer, lateral lig. reconstruction
23	45	left	posttraumatic	60	115	
	46	right	posttraumatic	69		
24	47	left	rheumatoid	72	122	PATL **^b^**
	48	right	rheumatoid	75		PATL **^b^**
25	49	left	posttraumatic	75	126	
	50	right	posttraumatic	79		
26	51	left	rheumatoid	85	149	PATL**^b^**
	52	right	rheumatoid	74		PATL **^b^**
mean (SD)				89 (22)	159 (35)	

**^a^** patient with additional bilateral total knee replacement.

**^b^** PATL: percutaneous Achilles tendon lengthening.

**^c^** MDCO: medial displacement calcaneal osteotomy.

**^d^** double-hindfoot AD: subtalar and talonavicular arthrodesis.

### Prosthesis and surgical technique

The HINTEGRA (Newdeal SA, Lyon, France) is an unconstrained 3-component system that provides intrinsic stability in the coronal plane (e.g. against eversion-inversion) (Hintermann et al. 2004, Barg et al. 2010a). Primary stability of the tibial component is obtained by screw fixation and 6 pyramidal peaks, while primary stability of the talar component is obtained by press-fit and screw fixation (cases 1–16). Since April 2003, 2 pegs instead of screws have been used for fixation of the talar component (cases 17–52). In the case of malalignment and concomitant osteoarthritis of the adjacent joints, additional surgeries (1-stage procedures) were performed before prosthetic implantation: osseous procedures in 12 ankles and soft tissue procedures in 24 ankles (Table 1). The double hindfoot arthrodesis (talonavicular and subtalar arthrodesis) was performed as described previously (De Wachter et al. 2007). The mean operative time was 2.6 h. The tourniquet time exceeded 2 h in 6 ankles (121−137 min). A single-dose of cefuroxime (1.5 g intravenously) was given preoperatively. All patients received subcutaneous low-molecular-weight heparin starting 12 h preoperatively and continuing daily for 6 weeks.

After surgery, a well-padded short-leg splint was used to hold the foot in a neutral position. After 24 h, the drains (without suction) were removed. In all cases, the drain production was less than 150 mL. After 2 days, the dressing and splint were removed, and a short leg walking cast was applied. 3–4 days after surgery, when the wound was dry, the cast was changed to a stable walker (VACOped; OPED AG, Cham, Switzerland). The duration of mobilization with a walker was 6 weeks (or 8 weeks when additional procedures such as adjacent joint fusion were performed). Full weight bearing in the stable walker was allowed as tolerated in all patients (with and without additional surgical procedures). Active and passive motion and manual lymphatic drainage were performed in all patients to support the recovery of soft tissues during the first 6 weeks. A rehabilitation program was continued after disuse of the walker—for at least 4 months, including walking exercises, stretching, and strengthening of the triceps surae. Patients also received instructive training in ankle motion and balance/proprioception. Low-level sports activities (e.g. hiking, swimming, biking, and golfing) were recommended and other sports activities were allowed (e.g. jogging, tennis, and downhill skiing). All patients were instructed to avoid contact sports or activities that involved jumping.

### Clinical examination

All patients were seen pre- and postoperatively in our outpatient clinic by 2 independent reviewers who did not perform the operations. The clinical examination involved assessment of ankle alignment and range of motion (ROM) with the patient standing (passive ROM activity), and assessment of ankle stability with the patient sitting. The ROM was determined clinically with a goniometer along the lateral border of the leg and foot.

Patients rated their pain on a visual analog scale (VAS) of 0 points (no pain) to 10 points (maximal pain). They also indicated their level of function in daily activities (e.g., walking and climbing stairs) and their satisfaction with the procedure (modified Coughlin rating for category scale: very satisfied, satisfied, partially satisfied, or not satisfied) (Coughlin 1990). In addition, we calculated the American Orthopaedic Foot and Ankle Society (AOFAS) hindfoot score (Kitaoka et al. 1994). Each patient's level of sports activity was documented preoperatively and during the latest follow-up using the following score: grade 0, none; grade 1, moderate; grade 2, normal; grade 3, high; and grade 4, elite (Valderrabano et al. 2006). In addition, all patients fully completed an SF-36 questionnaire on quality of life pre- and postoperatively (Ware and Sherbourne 1992).

### Radiographic measurements

Ankles were evaluated preoperatively based on weight bearing radiographs in two planes. In patients with obvious varus or valgus hindfoot deformity, a Saltzman view of the hindfoot was also taken (Saltzman and el Khoury 1995). Postoperative radiographic examinations were carried out using fluoroscopy to standardize the anteroposterior (AP) and lateral views of relevant components. Ankle radiographs were taken with the patients in a weight-bearing position.

**Figure 1.**
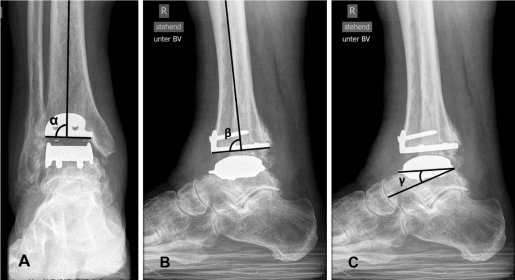
Angular measurements of prosthesis component positioning. α-angle (panel A) and β-angle (panel B) were measured between the longitudinal axis of the tibia and the articular surface of the tibial component in the AP and lateral views, respectively. γ-angle (C) was measured between a line drawn through the anterior shield and the posterior edge of the talar component and a line drawn along the center of the talar neck on the lateral view.

Angular and linear values were defined to digitally delineate alignment and component migration in the ankles (Hintermann et al. 2004) using the metric software system ImagicAccess (PIC Systems AG, Glattbrugg, Switzerland). The angular positions of the tibial and talar components were assessed from the α/β-angles (Hintermann et al. 2004) and from the γ-angle (Figure 1) (Lee et al. 2008). Loosening of the tibial component was defined as a change in position of the component's flat base by more than 2° relative to the long axis of the tibia and/or as a progressive radiolucency greater than 2 mm on the AP or lateral radiograph (Hintermann et al. 2004).

Loosening of the talar component, as seen on the lateral radiograph, was defined as subsidence into the talar bone by more than 5 mm, or a change in position of greater than 5° relative to a line drawn from the top of the talonavicular joint to the tuberosity of the calcaneus (Hintermann et al. 2004, Knecht et al. 2004). Evaluation of any minor change in position of the talar component on the AP radiograph was difficult, and it was not possible to evaluate radiolucencies beneath the talar component on either view. In cases with suspicion of loosening or subsidence, a CT scan or single-photon emission computed tomography (SPECT-CT) (Pagenstert et al. 2009) was performed. All radiographs were evaluated by two people and decisions were based on consensus.

### Statistics

A Kolmogorov-Smirnov test for normality was performed to determine whether data were normally distributed. Normally distributed data are presented as mean (SD). Non-normal data are presented as median (range). Kaplan-Meier survival analysis was performed with aseptic loosening of the tibial and/or talar component as the endpoint. Student's t-test and Mann Whitney rank sum test were used for comparison of data (normally and non-normally distributed data, respectively). Any p-value of ≤ 0.05 was considered to be statistically significant. Data were analyzed using SPSS software version 16.0 and also SigmaPlot 2004 (Systat Software Inc., San Jose, CA).

### Ethics

The study was conducted in accordance with the ethical standards of the responsible committee on human experimentation and in accordance with the Helsinki Declaration of 1975, as revised in 2000. The protocol was approved by the Ethics Committee of the University of Basel (reference no. 217/04), Switzerland. All participants provided informed written consent prior to surgery and study.

## Results

### Intraoperative and perioperative complications

As the surgery was performed with a tourniquet on the thigh, there was no intraoperative bleeding. No intraoperative complications were noted. Wound healing occurred within 2 weeks of the surgery, and was free of adverse events in all but 1 patient. There were no thromboembolic events after surgery.

### Postoperative complications

**Figure 2.**
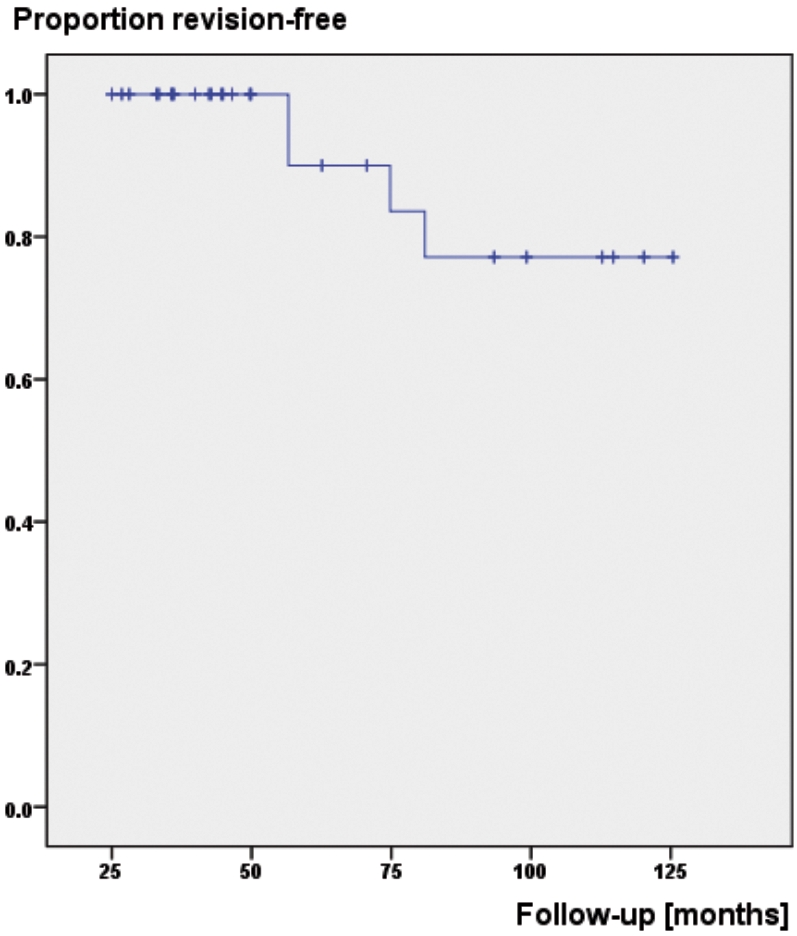
Kaplan-Meier survival curve with revision for aseptic loosening of the tibial and/or talar component as endpoint.

2 patients (patients 3 and 6) had revision of prosthetic components due to aseptic loosening (Table 2). The Kaplan-Meier survival analysis, with revision of any component for any reason as the endpoint, gave a survival rate of 91% at 5 years and 78% at 8 years (Figure 2).

**Table 2.** Postoperative complications and revision surgeries in 26 patients with simultaneous bilateral TAR

Case no. **^a^**	Postoperative complications	Surgery	Time of surgery **^b^** (years)	Results	Follow-up **^c^** (years)
5	aseptic loosening of both components	revision of both components (HINTEGRA)	6.7	both components are radiographically stable	2.4
6	aseptic loosening of both components	revision of both components (HINTEGRA), medial ligamentoplasty	6.2	both components are radiographically stable	2.9
11	aseptic loosening of talar component	revision of talar component (HINTEGRA), exchange of inlay	4.7	both components are radiographically stable	3.4
12	aseptic loosening of both components	revision of both components (HINTEGRA)	4.7	both components are radiographically stable	3.4
20	medial impingement	medial debridement	1.1	significant pain relief (VAS 2)	3.5
25	progressive hindfoot valgus deformity	medial displacement calcaneal osteotomy	2.5	normal hindfoot alignment	0.8

**^a^** Cases 5–50 have been reported previously (Barg et al. 2010b).

**^b^** since the primary surgery (TAR).

**^c^** after the revision surgery.

2 patients (cases 20 and 32) developed chronic pain due to medial impingement. In one patient (case 20), a medial debridement was performed 1.1 years after the primary surgery. In the other patient (case 32), local infiltrations led to pain relief; no revision surgery was necessary.

1 patient (case 25) developed a progressive painful valgus hindfoot deformity, which was treated by a medial displacement calcaneal osteotomy 2.5 years after the initial implantation of the prosthesis. At the follow-up, 1.4 years after re-alignment surgery, a neutral alignment was seen both clinically and radiographically. The patient was free of pain (VAS of 0).

1 patient (case 38) developed a painful cyst on the tibial side 2 years postoperatively. The patient refused open cyst debridement.

### Clinical results, radiographic outcome, and patient satisfaction

10 ankles were pain-free and 40 ankles had VAS scores of ≤ 2. Overall, there was substantial pain relief in all patients: the average pain score decreased from 7 (4−10) points to 2 (0−4) points (p < 0.001).

The AOFAS hindfoot score increased from 32 (SD 14) points preoperatively to 74 (SD 12) points postoperatively (p < 0.001).

Physical examination of the affected joints at the latest follow-up did not reveal any significant joint swelling, instability, or axial deformity of the affected joints. The average ROM increased from 28° (SD 12) preoperatively to 38° (SD 9) postoperatively (p < 0.001).

**Figure 3.**
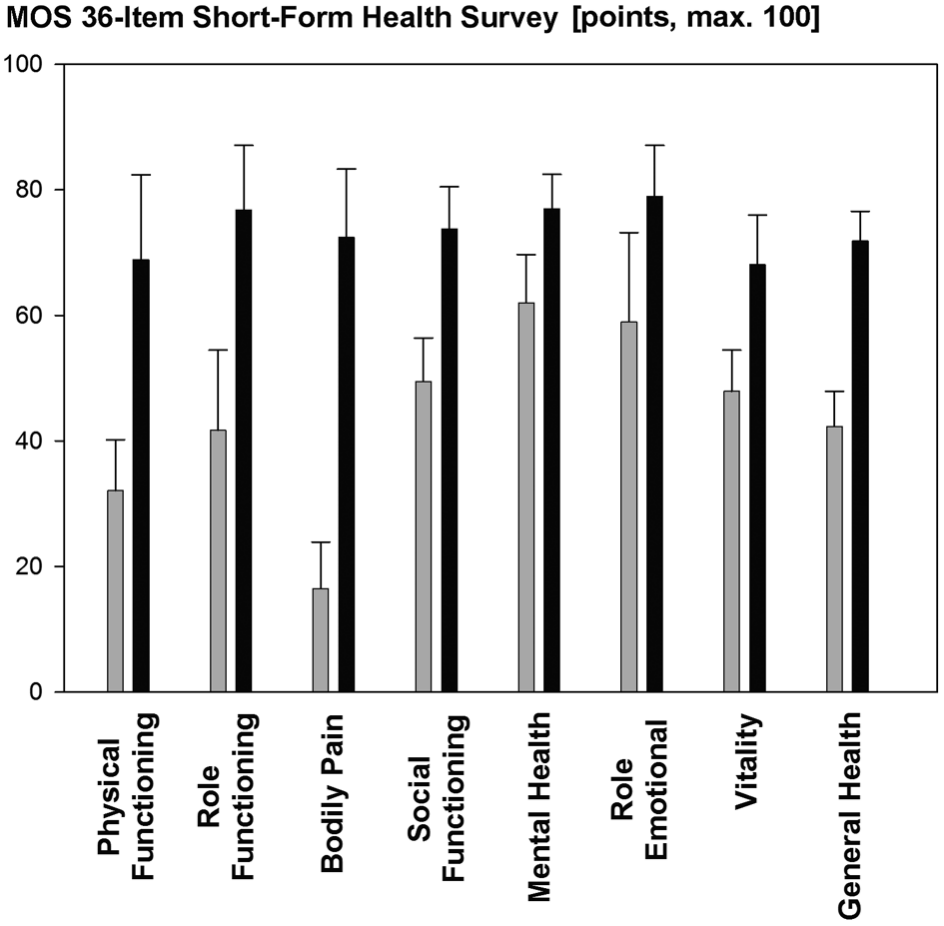
Preoperative (gray) and postoperative (black) quality of life for all patients assessed with the SF-36. Mean (SD). p < 0.001 for all pre- and postoperative comparisons.

All categories of the SF-36 score improved (p < 0.001) (Figure 3). The summarized components of the physical and mental outcomes score improved significantly from 35 (27–46) to 68 (59–90) (p < 0.001), and from 55 (37–68) to 73 (70–96) (p < 0.001), respectively.

Preoperatively, 5 patients had a normal level of sport activity and 3 patients had a moderate level. At latest postoperative follow-up, 8 patients had a normal level of sport activity, 6 patients had a moderate level, and 1 patient had a high level. 1 patient had a reduced level of sport activity (from normal to moderate).

At the final follow-up, both tibial and talar components were radiographically stable in all ankles. On the tibial side, no radiolucent lines were seen. The talar side was assessed for migration since the bone-component interface itself cannot be seen radiographically and loosening can only be inferred from potential migration of the implanted component. No migration of the talar component was detected. Bone fusion occurred in all patients with an additional arthrodesis. 8 ankles had mild heterotopic periarticular bone formations. The radiographic evaluation at the latest follow-up showed median α-, β-, and γ-angles of 90°, 85°, and 20°, respectively. There were no statistically significant intra-individual differences between the left and right sides.

At final follow-up, 7 patients were very satisfied with the outcome, 16 patients were satisfied, and 3 patients were satisfied with some reservation (two patients (cases 5/6 and 11/12) due to previous revisions and one patient (case 38) because of a painful cyst on the tibial side). All but one patient stated that he/she would choose the same operation again in a similar situation.

## Discussion

TAR is gaining acceptance as an option for patients with end-stage ankle osteoarthritis. However, there is limited information on the outcome of simultaneous bilateral TAR (Barg et al. 2010b, Karantana et al. 2010).

Karantana et al. (2010) published a series of 5 patients who had simultaneous bilateral TARs performed between 2002 and 2006 using the STAR prosthesis. The mean follow-up time was 4 (2–5) years. 2 patients had delayed wound healing. In 1 patient, a stress fracture of medial malleolus was seen 10 weeks postoperatively; it healed in an Aircast splint for 6 weeks. At the latest follow-up, all patients experienced substantial pain relief and good functional outcome, with excellent satisfaction (Karantana et al. 2010). Recently, we compared pain relief, quality of life, and functional outcome in 23 of the 26 patients reported in the current study (simultaneous bilateral TARs) with that for 46 matched unilateral TARs (Barg et al. 2010b). After 4 months, patients with simultaneous bilateral TAR had a higher level of pain and worse functional outcome and quality of life as assessed using AOFAS and SF-36 scores, respectively. However, the differences observed disappeared at the 1- and 2-year follow-ups (Barg et al. 2010b).

In the current study, all patients were followed for a minimum of 2 years to determine (1) intraoperative and postoperative complications, (2) the degree of postoperative pain relief, and (3) medium-term functional outcome.

There were no apparent intraoperative complications. However, intraoperative fractures of the medial or lateral malleoli and/or tendon/nerve laceration or injuries have been reported to be common (Saltzman et al. 2003, Lee et al. 2008). Wound healing complications may occur with an incidence of up to 28% (Whalen et al. 2010, Raikin et al. 2010). Only 1 of our patients had superficial infection, with delayed wound healing that healed after intravenous administration of antibiotics.

In 4 ankles, revision of prosthetic components was necessary because of aseptic loosening. 1 of the 2 patients had gouty arthritis and the other patient had rheumatoid arthritis. Reduced bone quality may be a reason for aseptic loosening. In addition, we believe that the prosthesis failure in these 2 patients was also related to the design. In both patients, the second-generation HINTEGRA prosthesis had been used. In this generation, fixation of the talar and tibial components was achieved using 2 screws. We believe that the talar component of the second-generation prosthesis is at greater risk of failure than the current third-generation prosthesis, which was introduced in April 2003. The third generation has a talar fixation with 2 pegs, which we believe may reduce the risk of aseptic loosening. The cumulative incidence of revision/reoperation for any reason was 6 of 26, which is similar to those that have been reported from other studies (Haddad et al. 2007, SooHoo et al. 2007, Gougoulias et al. 2010).

Postoperatively, all patients experienced an increase in ROM of 9° (1–25). However, in 28 ankles the increase in ROM postoperatively was less than 10°. These results are comparable to those reported in a recent systemic review of the literature, where there was a narrow range of improvement in ROM of 0–14° (Gougoulias et al. 2010).

The mental and physical disability associated with end-stage ankle osteoarthritis is at least as severe as that associated with end-stage hip osteoarthritis (Glazebrook et al. 2008). In the present study, all patients reported substantial postoperative improvement in quality of life regarding both the physical component and the mental component. This may partially explain the high grade of satisfaction in our patients.

More patients were active in sports after the surgery than before (57.7% vs. 30.8%). Naal et al. (2009) investigated habitual physical activity and sports participation in 101 patients who underwent TAR. In their study, where most patients had unilateral ankle osteoarthritis the levels of sport activity were similar before and after operation (two-thirds). We encourage patients to be normally or moderately active in sports after TAR because it has been shown that there is no association between increased physical activity levels and loosening of the prosthesis (Naal et al. 2009).

Our study has some limitations. Firstly, the senior surgeon who performed all the operations was involved with the design of the prosthesis, which may raise concerns about a conflict of interest. However, the clinical and radiographic evaluation was performed by observers who did not participate in any of the operations or in the design of the implant. Secondly, short intraoperative time, no intraoperative complications, and favorable postoperative outcomes may relate to the senior author's experience in performing TAR, particularly using this design of prosthesis. TAR remains a technically demanding procedure and should be limited to foot and ankle surgeons with considerable experience in TAR. Thirdly, the AOFAS score used for the clinical evaluation is not validated. Finally, follow-up in this study was limited to an average of 5 years.

In summary, we believe that simultaneous bilateral TAR can be safely performed in patients with bilateral end-stage ankle osteoarthritis. The clinical outcome and stability of the prosthesis are comparable to those reported for patients with unilateral TAR.
